# P-type ATPases: Many more enigmas left to solve

**DOI:** 10.1016/j.jbc.2023.105352

**Published:** 2023-10-12

**Authors:** Michael Palmgren

**Affiliations:** Department of Plant and Environmental Sciences, University of Copenhagen, Frederiksberg C, Denmark

**Keywords:** P-type ATPases, primary active transport, mechanism, evolution

## Abstract

P-type ATPases constitute a large ancient super-family of primary active pumps that have diverse substrate specificities ranging from H^+^ to phospholipids. The significance of these enzymes in biology cannot be overstated. They are structurally related, and their catalytic cycles alternate between high- and low-affinity conformations that are induced by phosphorylation and dephosphorylation of a conserved aspartate residue. In the year 1988, all P-type sequences available by then were analyzed and five major families, P1 to P5, were identified. Since then, a large body of knowledge has accumulated concerning the structure, function, and physiological roles of members of these families, but only one additional family, P6 ATPases, has been identified. However, much is still left to be learned. For each family a few remaining enigmas are presented, with the intention that they will stimulate interest in continued research in the field. The review is by no way comprehensive and merely presents personal views with a focus on evolution.

P-type ATPases are biological pumps with very different ligand specificities but have in common the same catalytic machinery (1, 2). The name P-type (from phosphorylation type) was coined on the basis of these pumps all being auto-phosphorylated during catalysis at a conserved aspartate residue (3). This review mainly focuses on evolutionary aspects of these pumps, as detailed structural comparisons have recently been presented elsewhere (4). First, it is worth noting the fascinating early history of research into P-type ATPases, which took off 70 years ago.

In 1953, Jens Christian Skou and Robert Post met in Wood’s Hole, MA, for the first time and their paths were to cross again in the years to come (5, 6). At Wood’s Hole, scientists interested in the functioning of the nervous system had come together and used giant axons from squids as test subjects (6). At the time, Na^+^ was known to be essential for the electrical impulses conducted by nerve axons. Back in Denmark, Jens Christian Skou isolated crab nerves and tested whether the addition of Na^+^ to membranes isolated from homogenates of the nerves stimulated their rates of ATP hydrolysis. The results were not clear, and it took a while to establish that to obtain reproducible results, K^+^ also had to be present in the preparation (described in ref. (6)). In 1957, Skou reported that he had identified an ATP hydrolytic activity in the membranes of crab nerves, which was dependent on the simultaneous presence of Na^+^ and K^+^ ions, and he concluded that the ATPase present in crab nerves exhibited a number of features predicted to be essential in enzymes that actively extrude Na^+^ from nerve cells (7). That same year, Robert Post, who after having given up working on squid axons had shifted to human erythrocytes, concluded that three Na^+^ ions were actively transported out of erythrocytes for every two K^+^ ions actively transported into these cells (8). In 1960, Post *et al.* demonstrated that an ATP hydrolytic activity like that observed by Skou in crab nerves (7) was present in erythrocyte membranes and the observed activity had nine characteristics that were the same as reported for the transport of K^+^ and Na^+^; most strikingly, both activities were inhibited by the cardiac glucoside ouabain (9). This body of work confirmed that the ATPase studied was part of a transport system for Na^+^ and K^+^*.* For his discovery of the Na^+^/K^+^-ATPase, Skou was awarded the Nobel Prize in 1997. The Na^+^/K^+^-ATPase was the first P-type ATPase to be identified.

After having studied cation-dependent ATP-ADP exchange kinetics of the Na^+^/K^+^-ATPase, Skou concluded that “an intermediate step in the breakdown of ATP is the formation of a phosphorylated enzyme in which the phosphate is bound to the enzyme by an energy-rich bond” (10). Indeed, using radioactively labeled ATP, it was subsequently shown that Na^+^ alone stimulated the incorporation of phosphate in the enzyme and that K^+^ had the opposite effect (11, 12, 13). It was found that the terminal γ-phosphate group of ATP forms an acyl phosphate bond to an aspartate residue in the amino sequence of the Na^+^/K^+^-ATPase (14) and also the Ca^2+^-ATPase from sarcoplasmic reticulum (15). This and other similarities caused Post *et al*. (15) to suggest that the two enzymes had evolved from a common ancestor.

Following the advent of molecular biology methods, the bacterial K^+^-ATPase KdpB was cloned in 1984 (16), and the following year animal sequences of Na^+^/K^+^-, H^+^/K^+^, and Ca^2+^-ATPases were published (17, 18, 19, 20). The catalytic subunit of P-type ATPases was found to be a membrane-embedded polypeptide of about 1000 residues (Fig. 1*A*) with the phosphorylated aspartate being in the signature sequence motif Asp-Lys-Thr-Gly-Thr (Fig. 1*B*). The plasma membrane ATPase of plants and fungi is a hydrogen ion pump and cloning of this enzyme from the yeast *Saccharomyces cerevisiae* revealed strong sequence similarity around the phosphorylatable aspartate residue identified in both the bacterial and animal pumps (21). Thus, it became evident that a large group of ATPases with a phosphorylated intermediate from all major domains of life had evolved from a common ancestor protein.

**Figure 1:**
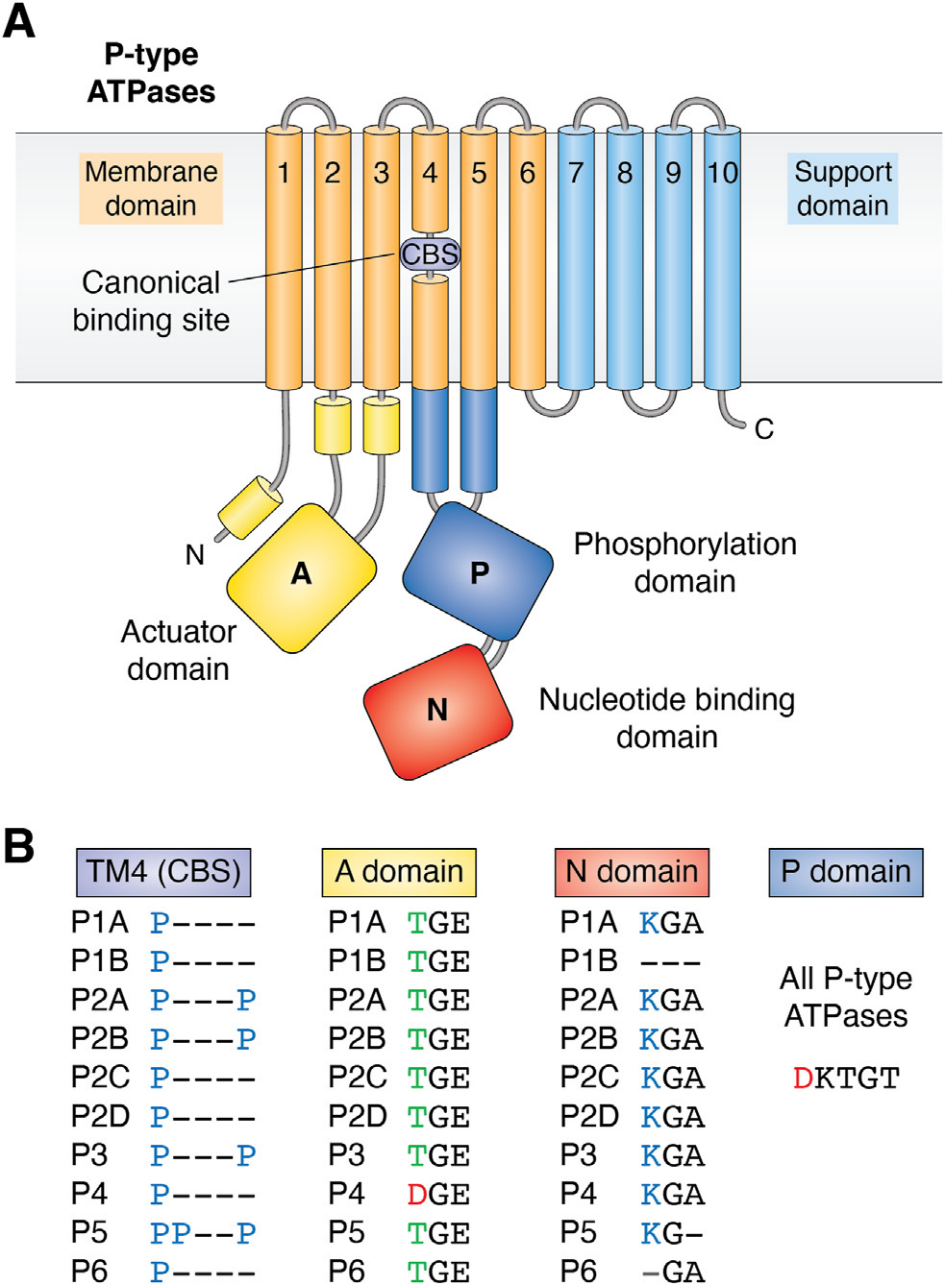
**Overview of domain organization in P-type ATPases**. *A*, the catalytic machinery consists of three cytosolic domains (A, P, and N) and two membrane-located domains (M and S). All P-type ATPases are phosphorylated and dephosphorylated during the catalytic cycle at an invariant aspartate residue (*red letter* in protein sequence) in the phosphorylation (P) domain (shaded *dark blue*). The phosphorylation reaction is carried out by the nucleotide-binding (N) domain (shaded *red*), which binds ATP and is an inbuilt protein kinase. The dephosphorylation reaction is carried out by the actuator (A) domain (shaded *yellow*), which is an in-built protein phosphatase. Phosphorylation and dephosphorylation of the pump cause conformational changes in the membrane domain (M) (*orange helices*) where the ligand(s) to be transported are bound. This domain comprises six transmembrane helices among which transmembrane helix 4 (TM4) is broken by one or more proline residues, which give room for a cavity and provide a saddle for the ligand to rest on. This ligand binding site is commonly referred to as the CBS. The support (S) domain (*light blue helices*) delivers structural support for the M domain and varies with respect to the number of helices and location at either the N- or C-terminal end. An autoinhibitory regulatory (R) domain may also be present at either terminal (not shown). *B*, conserved sequence motifs in P-type ATPase domains. The phosphorylatable aspartate (D) residue is present in a signature motif of P-type ATPases: DKTGT. The CBS in TM4 of the M domain results from helix-breaking proline (P) residues, the number of which varies from one to three depending on the P-type ATPase subfamily. The phosphatase motif in the A domain includes a negatively charged glutamate (E) residue in all pumps but in P4 ATPases also an aspartate (D) residue. The ATP binding site in the N domain includes a conserved lysine (K) residue, which is absent in P1B and P6 ATPases. CBS, canonical binding site; DKTGT, Asp-Lys-Thr-Gly-Thr.

First proposed by R. Wayne Albers (22) and modified by Post *et al.* (23), P-type ATPases follow an alternating access mechanism with two major conformations termed E1 and E2 (short for Enzyme1 and Enzyme2; the phosphorylated forms are denoted E1P and E2P, respectively). That a P-type ATPase can alternate between different conformations was supported by the observation of Peter Leth Jørgensen, a student of Skou, that degradation of the Na^+^/K^+^-ATPase by trypsin gave rise to two different patterns of proteolytic products depending on the presence Na^+^ or K^+^ (24). According to the so-called Post-Albers model, it is central for the mechanism of P-type ATPase that this pump alternates between inward-facing E1 and outward-facing E2 conformations (Fig. 2). The conformational changes are induced by phosphorylation of E1. E1P is spontaneously converted to E2P. Dephosphorylation of E2P is followed by the spontaneous conversion of E2 to E1. Phosphorylation and/or dephoshorylation is triggered by binding of the ligand(s) to be transported.

**Figure 2:**
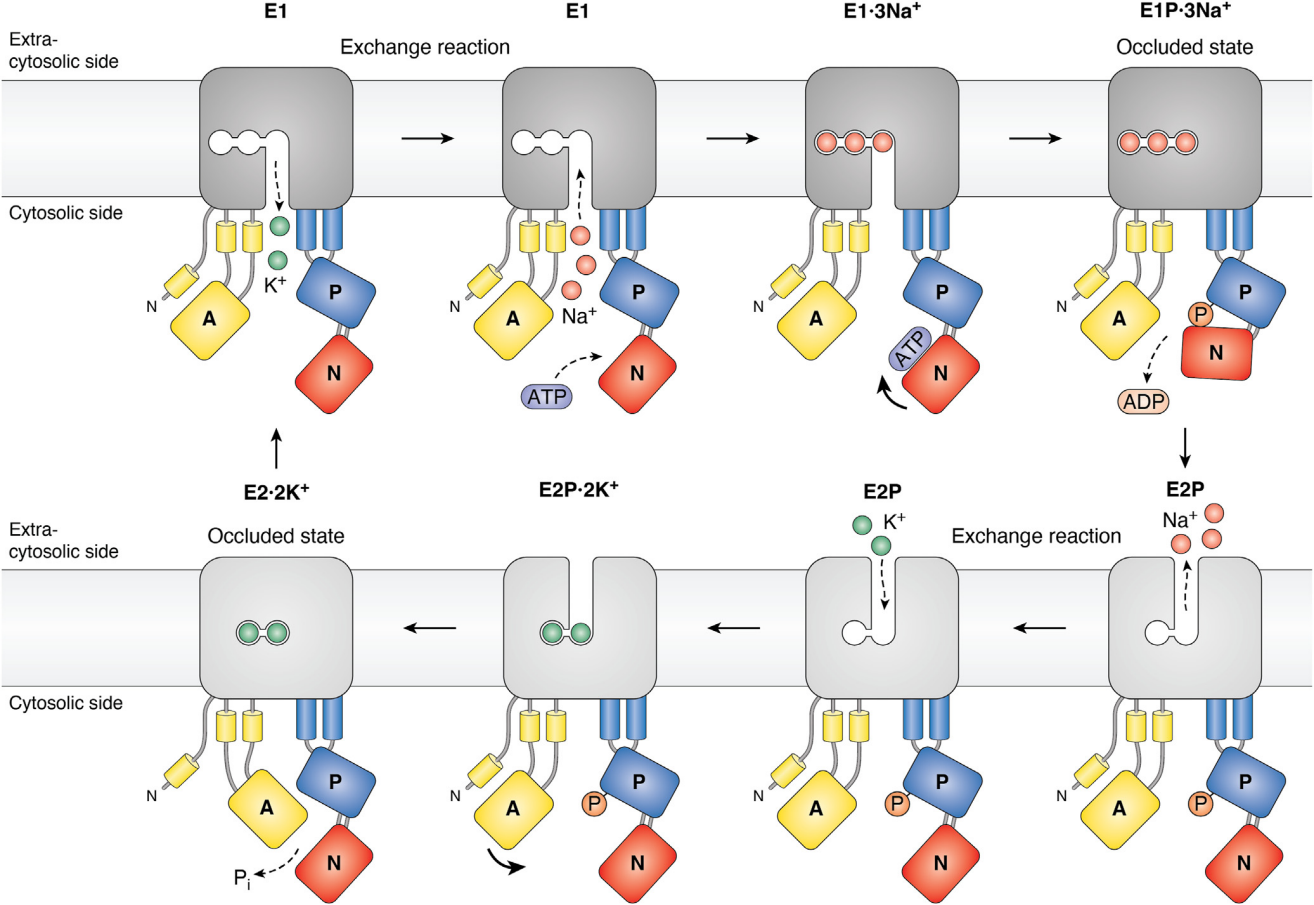
**Catalytic cycle and mechanism of a P-type ATPase with the Na**^**+**^**/K**^**+**^**-ATPase used as an example.** According to the Post-Albers scheme, which has been confirmed through numerous structures in different states, P-type ATPases alternate between two major conformations termed E1 and E2. The E1 conformation has one or more high-affinity membrane-located ion binding site(s) exposed to the cytosolic side of the membrane (in this case for Na^+^; the size of ions is enlarged for clarity). The site(s) have a low affinity for the counter-transported ligand (here K^+^). Phosphorylation of the pump by ATP causes a transition to the E2 conformation, which has the ion binding site(s) exposed to the extra-cytosolic side of the membrane. Now the site(s) have low affinity for the ligand to be exported (Na^+^) and high affinity for the counter-transported ligand (K^+^) and an exchange reaction takes place. Phosphorylation of the pump is triggered by binding of the (last in case there is more than one) ligand to be transported. Dephosphorylation is triggered by the (last) ligand to be counter-transported. In this way, coupling between ATP hydrolysis and transport is assured. During the reaction cycle, it is the cytosolic phosphorylation (P) domain that is phosphorylated. See text and Figure 1 for more details.

The first structure of a P-type ATPase, that of the sarcoplasmic reticulum Ca^2+^-ATPase, was published in a landmark paper by Toyoshima *et al*. in 2001 (25). Multiple structures of P-type ATPases in the E1 and E2 conformations are now available and for an update on structure–function relationships, the reader is referred to a recent excellent review (4). Taken together, the structures combined with evidence from mutagenesis experiments have revealed that P-type ATPases are divided into five domains: Three exposed to the cytosol and two embedded in the membrane (reviewed in ref. (1)) (Fig. 1*A*). The portion of P-type ATPases exposed to the cytoplasm consists of three catalytical domains: the phosphorylation (P) domain that gets phosphorylated during catalysis, the nucleotide binding (N) domain that as an in-built protein kinase phosphorylates the P domain, and the actuator (A) domain that serve as an in-built protein phosphatase to dephosphorylate the P domain. Transported ligand(s) are bound at binding sites in the middle of the plane of the membrane (M) domain. The M domain is supported by the S domain, which varies in composition between P-type ATPase families. Depending on the conformation, the ligand(s) is accessible to either one or the other side of the membrane (Fig. 2). By an alternating access mechanism, a high-affinity ligand-binding site facing one side of the membrane is transduced into a low-affinity site on the other side of the membrane. As each conformation has its own ligand specificity (or specificities), vectorial transport in both directions is possible during a single catalytic cycle (Fig. 2).

A phylogenetic analysis carried out in 1998 considered all available P-type ATPase sequences at that time, 254 in total, and revealed the existence of five major P-type ATPase families termed P1 to P5 (26). Due to a lack of computer power and suitable algorithms, the statistical analysis was limited and only based on 12 phylogenetic trees. Reanalysis of the same sequences, however, does result in essentially the same tree with strong statistical support at the nodes for most families (Fig. 3*A*). Some of the families are further divided into subfamilies. Characteristic for each family or subfamily is that they have a distinct substrate specificity (Fig. 3*B*). There is variation with respect to the presence of accessory subunits and the position and number of transmembrane helices in the S domain (Fig. 4). Two families (P4 and P5) are unique to eukaryotes where they are omnipresent. The evolutionary relationship between families remains uncertain as the nodes at the central parts of the unrooted tree lack statistical support.

**Figure 3:**
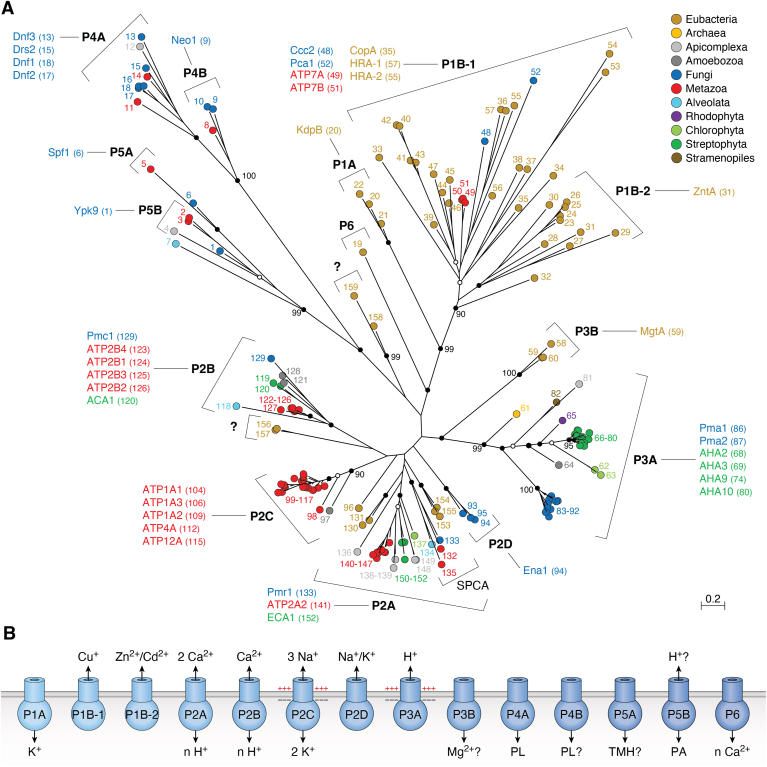
**Evolution and function of P-type ATPases.***A*, phylogenetic tree based on core sequences of 159 P-type ATPases. The sequences used and their numbers used for constructing the phylogenetic tree are the same as in Axelsen and Palmgren (1998). Clades that represent different P-type ATPase families and sub-families are named P1A to P6. A question mark indicates that it is uncertain to which family a sequence(s) belong. The tree was constructed using the maximum likelihood method using the program RAxML and Bayesian inference analysis using the program MrBayes as described (Palmgren, 2023). Shown is the best RAxML tree after 1000 bootstrap rounds. Number at major nodes indicate bootstrap values of ≥90. A separate Bayesian inference analysis was carried out, which resulted in a similar tree. The Bayesian inference analysis was run for 3,000,000 generations with a resulting average standard deviation of split frequencies at 0.01. *Black dots* at nodes in the RAxML tree indicate maximum statistical support (*p* = 1) in the Bayesian inference analysis; *open circles* at nodes indicate that *p ≥* 0.95. Names of some proteins from *Homo* sapiens, the yeast *Saccharomyces cerevisiae* and the plant *Arabidopsis thaliana* are given before numbers. Numbers refer to sequences as follows: 1, Q12697 Ypk9 (*Saccharomyces cerevisiae*); 2, Q27533 W08D2.5 (*Caenorhabditis elegans*); 3, Q21286 catp-5 (*C. elegans*); 4, Q04956 Probable cation-transporting ATPase 1 (*Plasmodium falciparum*); 5, P90747 catp-8 (*C. elegans*); 6, P39986 Spf1 (*S. cerevisiae*); 7, Q95050 TPA9 (*Tetrahymena thermophila*); 8, G5EBH1 tat-5 (*C. elegans*); 9, P40527 Neo1 (*S. cerevisiae*); 10, Q10309 neo1 (*Schizosaccharomyces pombe*); 11, Q7JPE3 tat-4 (*C. elegans*); 12, Q27720 Phospholipid-transporting ATPase (*P. falciparum*); 13, Q12674 Dnf3 (*S. cerevisiae*); 14, Q29449 ATP8A1 (*Bos taurus*); 15, P39524 Drs2 (*S. cerevisiae*); 16, Q09891 dnf2 (*S. pombe*); 17, Q12675 Dnf2 (*S. cerevisiae*); 18, P32660 Dnf1 (*S. cerevisiae*); 19, P9WPT1 ctpE (*Mycobacterium tuberculosis*); 20, P03960 KdpB (*Escherichia coli*); 21, P9WPU3 KdpB (*M. tuberculosis*); 22, P73867 KdpB (*Synechocystis* sp. PCC 6803 substr. Kazusa); 23, P94888 cadA (*Lactococcus lactis*); 24, Q60048 cadA (*Listeria monocytogenes*); 25, P30336 cadA (*Alkalihalophilus pseudofirmus*); 26, P37386 cadA (*Staphylococcus aureus*); 27, Q59998 ziaA (*Synechocystis* sp.); 28, Q59997 slr0797 (*Synechocystis* sp.); 29, Q59465 cadA (*Helicobacter pylori*); 30, P9WPS7 ctpG (*M. tuberculosis*); 31, P37617 zntA (*E. coli*); 32, P9WPT5 ctpC (*M. tuberculosis*); 33, P77871 copA (*H. pylori*); 34, P77868 HI_0290 (*Haemophilus influenzae*); 35, Q59385 copA (*E. coli*); 36, P05425 copB (*Enterococcus hirae*); 37, P37385 synA (*Synechococcus elongatus*); 38, P74512 Cation-transporting ATPase (*Synechocystis* sp.); 39, P9WPS3 ctpV (*M. tuberculosis*); 40, P46840 ctpB (*Mycobacterium leprae*); 41, P9WPU1 ctpA (*M. tuberculosis*); 42, P9WPT9 ctpB (*M. tuberculosis*); 43, P46839 ctpA (*M. leprae*); 44, Q59688 copA_3 (*Proteus mirabilis*); 45, P73241 pacS (*Synechocystis* sp.); 46, P37279 pacS (*S. elongatus*); 47, P77881 ctpA (*L. monocytogenes*); 48, P38995 Ccc2 (*S. cerevisiae*); 49, Q04656 ATP7A (*Homo sapiens*); 50, Q64535 Atp7b (*R. norvegicus*); 51, P35670 ATP7B (*H. sapiens*); 52, P38360 Pca1 (*S. cerevisiae*); 53, Q59207 fixI (*Bradyrhizobium diazoefficiens*); 54, P18398 fixI (*Rhizobium meliloti*); 55, Q59370 HRA-2 (*E. coli*); 56, P32113 copA (*E. hirae*); 57, Q59369 HRA-1 (*E. coli*); 58, P22036 mgtB (*Salmonella typhimurium*); 59, P0ABB8 mgtA (*E. coli*); 60, P36640 mgtA (*S. typhimurium*); 61, Q58623 MJ1226 (*Methanocaldococcus jannaschii*); 62, P54210 DHA1 (*Dunaliella acidophila*); 63, P54211 PMA1 (*D. bioculata*); 64, P54679 patB (*Dictyostelium discoideum*); 65, O04956 Plasma membrane ATPase (*Cyanidium caldarium*); 66, Q43178 PHA2 (*Solanum tuberosum*); 67, Q03194 PMA4 (*Nicotiana plumbaginifolia*); 68, P19456 AHA2 (*A. thaliana*); 69, P20431 AHA3 (*A. thaliana*); 70, Q43131 Plasma membrane ATPase (*Vicia faba*); 71, Q43275 zha1 (*Zostera marina*); 72, Q43271 MHA2 (*Zea mays*); 73, P93265 PMA (*Mesembryanthemum crystallinum*); 74, Q42556 AHA9 (*A. thaliana*); 75, Q43002 OSA2 (*Oryza sativa*); 76, Q43243 MHA1 (*Z. mays*); 77, Q43001 OSA1 (*O. sativa*); 78, Q42932 Plasma membrane ATPase (*N. plumbaginifolia*); 79, Q43106 BHA-1 (*Phaseolus vulgaris*); 80, Q43128 AHA10 (*A. thaliana*); 81, P12522 H1B (*Leishmania donovani*); 82, A0A7S3XUR3 Plasma membrane ATPase (*Heterosigma akashiwo*); 83, P24545 PMA1 (*Zygosaccharomyces rouxii*); 84, P28877 PMA1 (*Candida albicans*); 85, P49380 PMA1 (*Kluyveromyces lactis*); 86, P05030 Pma1 (*S. cerevisiae*); 87, P19657 Pma2 (*S. cerevisiae*); 88, Q92446 PCA1 (*Pneumocystis carinii*); 89, P28876 PMA2 (*S. pombe*); 90, P09627 PMA1 (*S. pombe*); 91, P07038 pma-1 (*Neurospora crassa*); 92, Q07421 PMA1 (*Ajellomyces capsulatus*); 93, P22189 cta3 (*S. pombe*); 94, P13587 Ena1 (*S. cerevisiae*); 95, P78981 Z-ENA1 (*Zygosaccharomyces rouxii*); 96, P73273 ziaA (*Synechocystis* sp.); 97, Q76P11 ionA (*D. discoideum*); 98, G5EFV6 catp-4 (*C. elegans*); 99, P35317 ATP1A (*Hydra vulgaris*); 100, P28774 Na^+^/K^+^-ATPase alpha-B (*Artemia franciscana*); 101, Q27461 eat-6 (*C. elegans*); 102, P13607 Atpalpha (*Drosophila melanogaster*); 103, Q27766 Na^+^/K^+^-ATPase alpha (*Ctenocephalides felis*); 104, P05023 ATP1A1 (*H. sapiens*); 105, P05025 ATP1A (*Tetronarce californica*); 106, P13637 ATP1A3 (*H. sapiens*); 107, Q92030 atp1a1 (*Anguilla anguilla*); 108, P25489 atp1a1 (*Catostomus commersonii*); 109, P50993 ATP1A2 (*H. sapiens*); 110, Q64541 Atp1a4 (*R. norvegicus*); 111, P17326 Na^+^/K^+^-ATPase alpha-A (*A. franciscana*); 112, P20648 ATP4A (*H. sapiens*); 113, Q92126 atp4a (*Xenopus laevis*); 114, Q64392 ATP12A (*Cavia porcellus*); 115, P54707 ATP12A (*H. sapiens*); 116, P54708 Atp12A (*R. norvegicus*); 117, Q92036 ATP12A (*Rhinella marina*); 118, Q27829 Plasma membrane calcium ATPase (*Paramecium tetraurelia*); 119, P93067 Calcium-transporting ATPase (*Brassica oleracea*); 120, Q37145 ACA1 (*A. thaliana*); 121, Q27642 Calcium-transporting ATPase (*Entamoeba histolytica*); 122, Q64542 Atp2b4 (*R. norvegicus*); 123, P23634 ATP2B4 (*H. sapiens*); 124, P20020 ATP2B1 (*H. sapiens*); 125, Q16720 ATP2B3 (*H. sapiens*); 126, Q01814 ATP2B2 (*H. sapiens*); 127, G5EFR6 mca-1 (*C. elegans*); 128, P54678 patA (*D. discoideum*);129, P38929 Pmc1 (*S. cerevisiae*); 130, P9WPS9 ctpF (*M. tuberculosis*); 131, P37367 pma1 (*Synechocystis* sp.); 132, Q64566 Atp2c1 (*R. norvegicus*); 133, P13586 Pmr1 (*S. cerevisiae*); 134, Q95022 CppA-E1 (*Cryptosporidium parvum*); 135, Q27724 PfATPase4 (*P. falciparum*); 136, Q95060 TVCA1 (*Trichomonas vaginalis*); 137, P54209 CA1 (*Dunaliella bioculata*); 138, O09489 Calcium-transporting ATPase (*Leishmania amazonensis*); 139, P35315 TBA1 (*Trypanosoma brucei brucei*); 140, Q27779 SMA1 (*Schistosoma mansoni*); 141, P16615 ATP2A2 (*H. sapiens*); 142, P70083 atp2a1 (*Makaira nigricans*); 143, Q92105 ATP2A1 (*Pelophylax lessonae*); 144, Q64578 Atp2a1 (*Rattus norvegicus*); 145, P18596 Atp2a3 (*R. norvegicus*); 146, P22700 SERCA (*D. melanogaster*); 147, P35316 SERCA (*A. franciscana*); 148, Q08853 ATP6 (*P. falciparum*); 149, Q27764 YEL6 (*Plasmodium yoelii*); 150, Q42883 LCA1 (*Solanum lycopersicum*); 151, O04938 Ca^2+^-ATPase (*O. sativa*); 152, P92939 ECA1 (*A. thaliana*); 153, Q59999 sll0672 (*Synechocystis* sp.); 154, P37278 pacL (*S. elongatus*); 155, P74062 slr0822 (*Synechocystis* sp.); 156, P78036 pacL (*Mycoplasma pneumoniae*); 157, P47317 pacL (*Mycoplasma genitalium*); 158, P9WPS5 ctpI (*M. tuberculosis*); 159, P96271 ctpH (*M. tuberculosis*). Scale bar, 0.2 amino acid substitutions per site. *B*, overview of P-type ATPase families and transported ligands. Domain and subunit organization are not shown. For each subfamily is shown the ligand transported, the number of ligands transported per ATP hydrolyzed, and the direction of transport. Not shown are subunit and transmembrane helices. A subgroup of P2A ATPases, secretory pathway Ca^2+^-ATPases (marked SPCA in Fig. 3*A*), only transport one Ca^2+^ per ATP hydrolyzed. Depending on the stoichiometry of transport, several P-type ATPases can be electrogenic. P2C and P3A ATPases are highly electrogenic and maintain plasma membrane potentials that are negative on the cytosolic side of the membrane. Abbreviations: PA, polyamines; PL, phospholipids; TMH, transmembrane helices.

**Figure 4:**
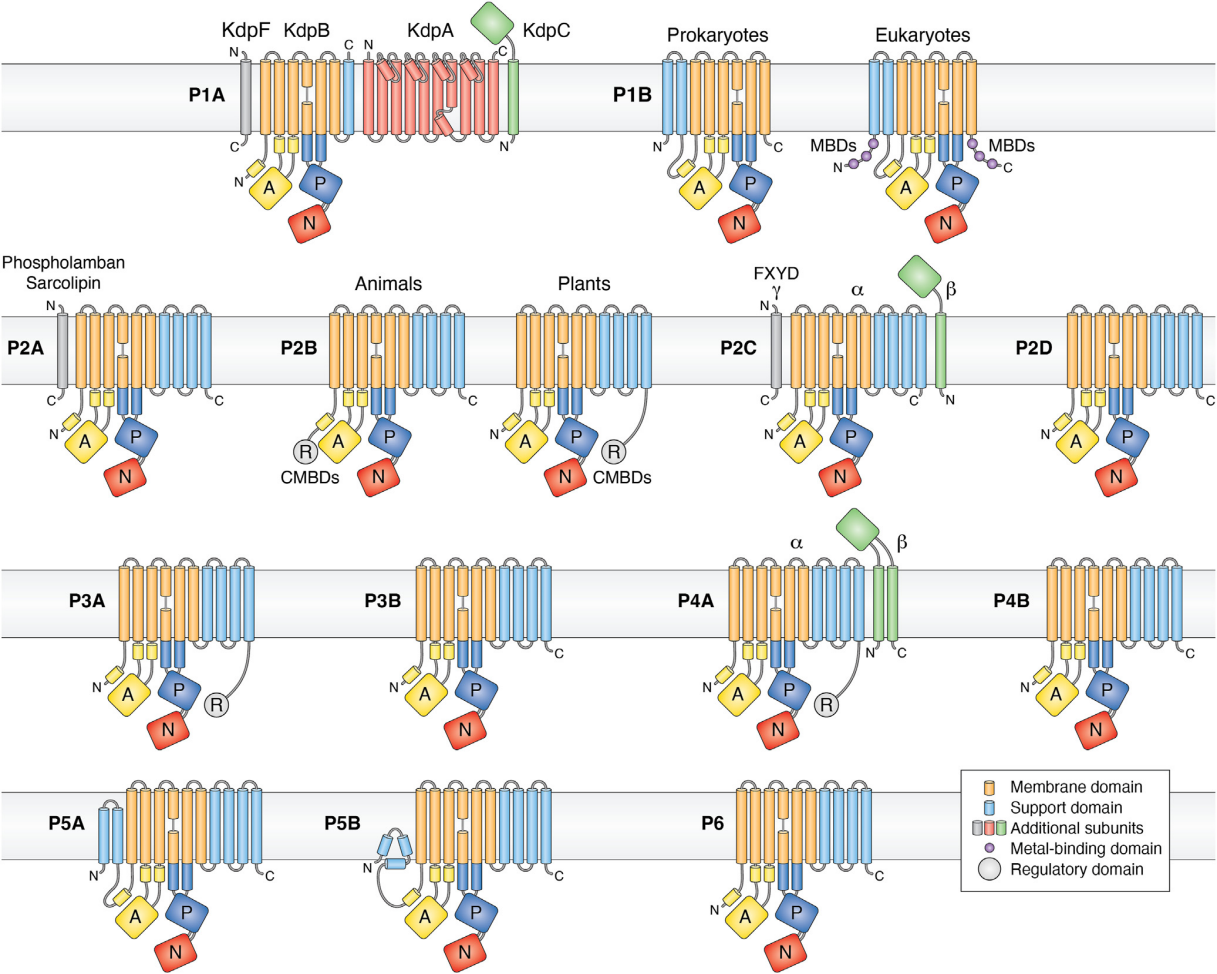
**Overview of the organization of transmembrane helices and accessory subunits in P-type ATPase family members**. All P-type ATPases have in common essentially the same catalytic machinery, which comprises three major cytosolic domains (A, P and N) and a membrane-embedded domain (M) composed of six transmembrane helices (shaded *orange* in the figure). Depending on the P-type ATPase family, additional transmembrane helices serve a support (S) function (shaded *blue*). The number of transmembrane helices vary from seven in P1A ATPases to 12 in P5A ATPases. In addition to the catalytic subunit, some families have additional subunits. Animal P2A ATPases may have a small hydrophobic subunit with a single transmembrane helix (phospholamban and sarcolipin) and P4A ATPases have a β subunit with two membrane helices with a heavily glycosylated domain facing the extra-cytosolic side of the membrane. P2C ATPases have a glycosylated β subunit with a single transmembrane helix and, in addition, can have a small hydrophobic subunit with a single transmembrane helix (the γ subunit and other subunits being members of the FXYD protein family). P1B ATPases have four subunits in total, one of which (KdpA) is a modified K^+^ channel protein. Some families have extended terminal autoinhibitory domains that serve a regulatory (R) function. The N-termini of eukaryotic P2C, P3A, and P4A ATPases and the C-terminus of eukaryotic P2C ATPases have also been implicated in the regulation of pump activity (not shown here). CMBDs, calmodulin-binding domains; MBD, metal-binding domains.

This review represents a personal reflection on mysteries that remain in the field of P-type ATPases. I highlight one or two unsolved problems specific to each P-type ATPase family. Many other enigmas remain in this area of research and, regretfully, space limitations prohibit me from discussing all of them.

## P1A ATPases: are they advanced or primitive?

P1A ATPases form heterotetrameric complexes of four subunits, KdpA, KdpB, KdpC, and KdpF, which together function as a high-affinity K^+^ uptake system (27). KdpA is a K^+^ channel that is connected to KdpB, the catalytic subunit of the P1A ATPase, whereas KdpC and KdpF are accessory subunits. KdpC was previously believed to be in the cytosol but is now known to be exposed extracellularly like the β-subunits of P2C and P4A ATPases. P1A ATPases are only found in prokaryotes and could therefore be considered primitive. Indeed, among P-type ATPases, they have the simplest catalytic subunit, KdpB, with only seven transmembrane helices (other P-type ATPases have from 8 to 12 transmembrane helices), yet they have the most complicated tertiary structure of any P-type ATPase. Strikingly, these pumps have the highest ligand affinity of any other P-type ATPases and possibly also the highest degree of ligand specificity.

**Figure 5:**
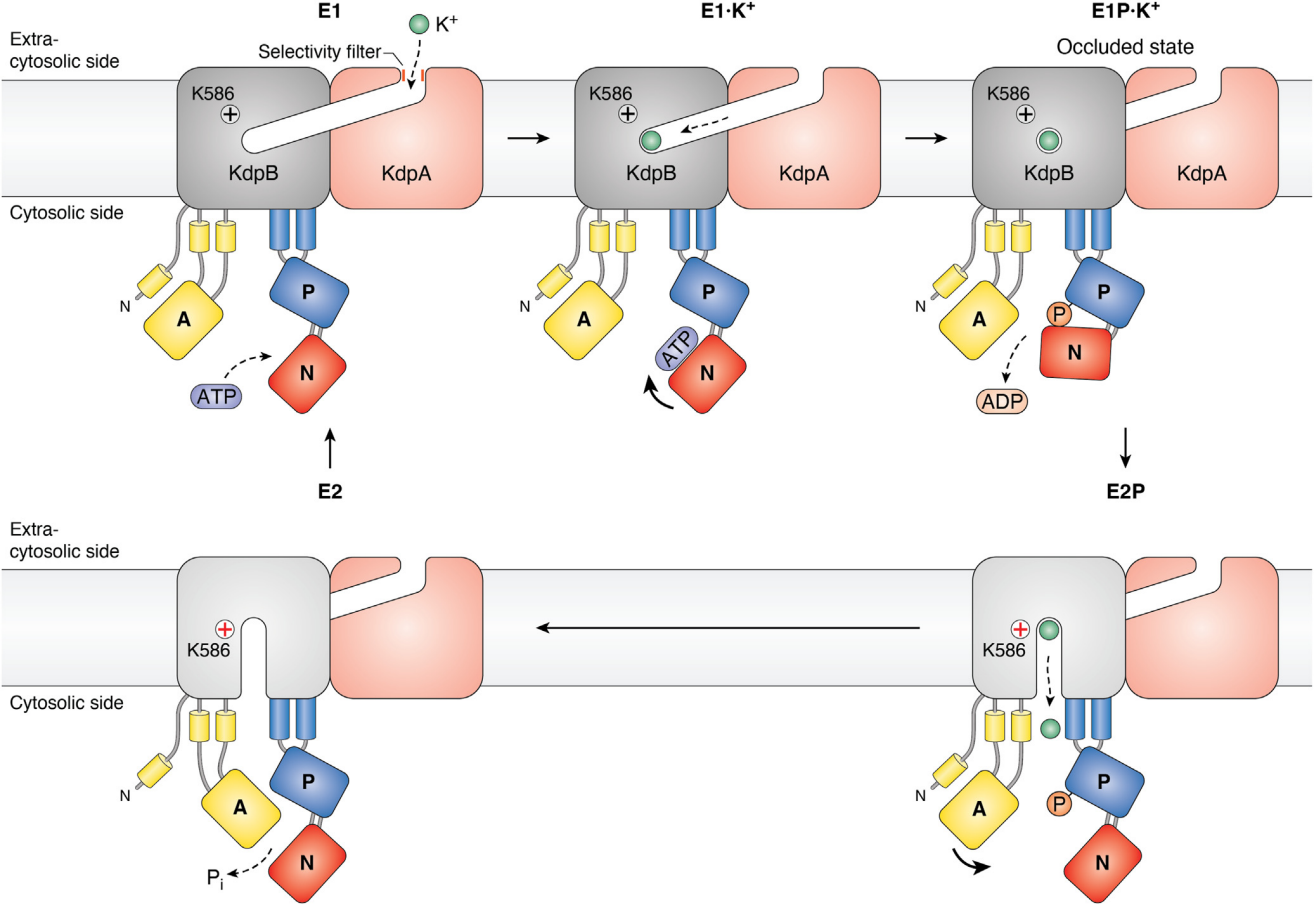
**Variations on a theme: the transport cycle of P1A ATPases.** P1B ATPases import K^+^ ions. The complex is composed of four subunits, only two of which (KdpB and KdpA) are shown here. K^+^ enters the complex through the selectivity filter (a) of KdpA, which is a modified K^+^ channel and moves through a tunnel that connects KdpA to the catalytic subunit KdpB. When K^+^ enters and binds to the canonical binding site in KdpB, the pump is phosphorylated from ATP. This phosphorylation causes the pump to transition to the E2P conformation and the K^+^ ion diffuses into the cell through a half-channel. Whether this half-channel is open in all conformations or only in the E2 state is difficult to ascertain due to the limited resolution of the structures. Dephosphorylation and return to the E1 conformation occur spontaneously possibly due to the action of Lys-856, which functions as a built-in cation. See text for more details.

It was first considered that KdpB may not bind K^+^ itself but rather functions to deliver energy for high-affinity transport of K^+^ through KdpA (28). Strikingly, the elucidation of the crystal structure of the *Escherichia coli* KdpFABC complex revealed the presence of a tunnel leading from KdpA to KdpB (29) (Fig. 5). A K^+^ ion was seen in the selectivity filter of KdpA and a water molecule was identified in a cavity in KdpB that is formed by an unwound part of transmembrane helix 4 (TM4), which includes a conserved proline residue, and resembles the canonical ion binding site of P-type ATPases (29). The tunnel could be seen to connect the K^+^ ion binding site in KdpA with the water site in KdpB. Although no densities could be observed in the tunnel, it was proposed that the water-filled tunnel could facilitate charge transfer *via* a proton wire between these two sites (29). Thus, binding of H^+^ to the canonical ion binding site in KdpB would trigger phosphorylation and a conformational change that would allow K^+^ to be released from KdpA to the cytoplasm (29).

Strikingly, in new structures of KdpFABC, non-protein densities were observed in the intersubunit tunnel and were suggested to represent K^+^ (30). At the interface between the two subunits, the tunnel was restricted by several residues, which suggested the presence of an entrance gate for K^+^ towards the ion binding site in KdpB (30). Structures of four conformational states of the KdpFABC demonstrated that KdpB undergoes conformational transitions between E1 and E2 states like other P-type ATPases whereas the other subunits remain static (30, 31). Furthermore, the presence of non-protein densities in the tunnel as well as in the KdpB cavity likely to represent K^+^ could be confirmed (31).

To confirm whether the densities in the tunnel represented water molecules or rather K^+^, cryo-EM structures of KdpFABC were obtained in the presence of high Rb^+^ (32). Rb^+^ is an analog of K^+^ but with a higher atomic number and thus able to scatter electrons more strongly. KdpFABC is strictly selective for K^+^ and does not allow the transport of other alkali cations such as Rb^+^ or Na^+^, but this problem was solved by employing a mutant of version of KdpA that allows for the passage of Rb^+^ through the selectivity filter of KdpA. In this way, Silberberg *et al*. (32) could obtain cryo-EM structures of KdpFABC with densities in the intersubunit tunnel that could be assigned to Rb^+^. In molecular dynamics simulations, two distinct energy minima were identified; the first at the selectivity filter and the second at the constricted part of the tunnel at the interface between KdpA and KdpB (32). Taken together, it now seems clear that the K^+^ enters KdpA *via* a selectivity filter from which K^+^ is funneled through a tunnel toward KdpB.

A simple mechanism serves to inhibit KdpB when K^+^ has become sufficient. Thus, when cells are experiencing high K^+^, the KdpB catalytic subunit is phosphorylated within less than 2 minutes at a residue, the phosphorylation of which causes an immediate block of catalytic turnover (33). This residue is the serine in the Thr-Gly-Glu-Ser (TGES) motif of the A domain that serves as a built-in protein phosphatase during catalytic phosphoenzyme turnover (29). The locked conformation of the serine-phosphorylated form is divergent from any other conformation in the canonical Post-Albers cycle and has been denoted E1P-tight (34).

In models based on comparison with the cytoplasmic headpiece structures of the P2A Ca^2+^-ATPase SERCA, K^+^ binds to KdpB in the E1 conformational state. This is striking and in contrast to other P-type ATPases since access to the ion binding site is *via* the tunnel that is connected to the extracytosolic side. The binding of K^+^ then triggers phosphorylation from ATP at the catalytic aspartate (Asp-307 in *E. coli* KdpB; ref. (35)) and the formation of the E1P phosphorylated state. In the subsequent spontaneous conformational change to the E2P state, the K^+^ in the high-affinity K^+^ binding site facing the tunnel is transferred to a low-affinity binding site facing the cytosol, in this way allowing for the release of K^+^. Subsequent dephosphorylation of E2P causes conformational change back to E1 (29, 30, 31, 32). If the binding of K^+^ causes phosphorylation, then what causes the dephosphorylation event?

Many P-type ATPases couple transport of an ion in one direction to transport of an ion in the other direction, and therefore the reaction cycle cannot be completed without counterions, as illustrated by the reaction cycle of the Na^+^/K^+^-ATPase (a P2C ATPase) (Fig. 2). However, some families complete the reaction cycle without countertransport and instead exploit an in-built counterion. Thus, a positively charged residue in TM5 (typically a lysine residue) is supposed to trigger dephosphorylation in P1B-2, P3A, and P4A ATPases (36, 37, 38, 39). Likewise, in the *E. coli* P1A ATPase, Lys-586, which is situated in the canonical ion binding site, may serve as a built-in counter-ion that triggers spontaneous dephosphorylation immediately following K^+^-induced phosphorylation (32).

Based on the advanced mechanism of high-affinity uptake of K^+^ it could be argued that P1A ATPases are the most advanced P-type pumps known. Apparently, KdpB is present in most, if not all, eubacterial phylae, whereas it is much less common in Archaea and not at all found in eukaryotes. Eukaryotes also depend on K^+^. How did it come to pass those eukaryotes lost such an advanced high-affinity K^+^ uptake system? One explanation could be that P1A ATPases evolved in Eubacteria after the evolution of eukaryotes and represent one of the most recent steps in P-type ATPase evolution.

## P1B ATPases: are eukaryotic metal pumps transceptors?

P1B ATPases export heavy metals such as zinc, cadmium, silver, copper, and lead (40, 41, 42). They are by far the most diverse group, which could indicate that they have evolved over a very long period. They are found in every living organism from prokaryotes to eukaryotes and therefore must be essential for life. Compared to Ca^2+^, which is universally abundant, heavy metals are seldom present in high concentrations. Several heavy metals such as zinc and copper are essential for life but above certain concentrations, they become toxic, implying that their cellular concentrations must be kept within a narrow range. Uptake of divalent cations is energetically very favorable due to the inside negative plasma membrane potential present in most organisms, but their export requires active transport, which explains why P1B ATPases are all export pumps, either exporting heavy metals out of the cell or into intracellular compartments.

P1B ATPases can be divided into two major groups, P1B-1 ATPases, which transport Cu^+^ and Ag^+^, and P1B-2 ATPases, which transport Zn^2+^ and Cd^2+^, and in addition, some minor sister clades have been described (40). P1B-3 ATPases were proposed to transport Cu^2+^ but in fact transport Cu^+^ and are now classified as P1B-1 ATPases (43). The structure of a prokaryotic P1B-1 ATPase in the inward-facing E1 conformation has localized the Cu^+^ ion to an uptake site in the membrane domain close to the unwound transmembrane helix 4 (TM4), the canonical ion binding site of all P-type ATPases (44) (Fig. 1*A*).

Animals only have P1B-1 ATPases whereas plants have both P1B-1 and P1B-2 ATPases. Zn^2+^ is an essential element and why Zn^2+^ pumps are dispensable in animals but not in plants is not well understood. One hypothesis is that they were acquired by plants by horizontal gene transfer from chloroplasts of cyanobacterial origin (45). Thus, in phylogenetic trees of P1B ATPases, eukaryotic sequences can often be seen embedded within prokaryotic clades. In such cases, where the structure of a phylogenetic tree does not follow the generally accepted evolution of species, horizontal gene transfer may have taken place (46).

P1B-4 ATPases, which constitute a small subgroup of P1B-2 ATPases (40), provide one example. In the phylogenetic tree in Figure 3*A* they are only represented by a single sequence from *Synechocystis* sp. (#28, Q59997). The structure of a eubacterial P1B-4 ATPase has been solved and in this pump, a histidine residue serves as the internal counterion (39). In eukaryotes, these pumps are found only in green algae and land plants (Plantae). Plant P1B-4 sequences show the highest homology to sequences from the eubacterial phylum Chlamydiae (45, 46). This would imply that P1B-4 ATPases were either present in the last common eukaryotic ancestor and subsequently lost in all kingdoms but plants, or that they were acquired at a later stage in plants by horizontal gene transfer, possibly from Chlamydiae (45). This scenario is in line with the hypothesis that the ancestor of Plantae was phagotrophic and consumed bacteria; it retained cyanobacteria (that ultimately became chloroplasts) and, eventually, portions of the DNA of bacteria that had been digested became integrated into the host genome (47).

But why were P1B-4 ATPases required by plants and what are their physiological roles in plants? P1B-4 ATPases have rather broad substrate specificities and bacterial P1B-4 ATPases have been shown to export Fe^2+^ when the iron load gets too high (48, 49, 50, 51, 52). The sole member in land plants, HMA1, localizes to the chloroplast envelope (53) where its role remains controversial. This protein was first proposed to import Cu into chloroplasts and later shown to scavenge Cu and Zn from the chloroplast to the cytosol, when these metals are required elsewhere in the plant (54), and to export Cd when this metal reaches toxic levels in chloroplasts (55). Iron is central for photosynthesis. However, the function of HMA1 in iron redistribution within the plant cell remains an open question.

A marked difference between P1B sequences from prokaryotes and eukaryotes is that those of the latter are much longer at their termini. Thus, the extensions are not in the catalytic domains or transmembrane-spanning helices but almost exclusively in the N- and C-terminal regions. This would suggest that the add-ons do not participate in the canonical transport mechanisms as such but rather have regulatory roles.

The P1B-1 ATPase ATP7A (or the Menkes Disease protein) of animals is exporting copper into the trans-Golgi network to deliver copper to copper-dependent enzymes in the secretory pathway (56). In this pump, the terminal domains play a role in regulated targeting to other membranes. Thus, in conditions of elevated copper ATP7A traffics to the plasma membrane and becomes a cellular export pump (57), and in polarized epithelial cells of the gut and kidney, ATP7A is targeted to the basolateral membrane in response to elevated copper (58). The copper-induced trafficking of ATP7A to the plasma membrane involves phosphorylation of Ser residues in the N- and C-terminal cytoplasmic domains (59). From the plasma membrane, the pump can be recycled back to the trans-Golgi network in a process where two di-leucine motifs in the C-terminal domain serve as targeting signals (60, 61). Sensing of copper is not necessarily linked to restoration of copper levels and could have other functions in cells that have low fluxes of copper. For example, in adipocytes and neurons, ATP7A-dependent transport of copper into the secretory pathway is required for cellular differentiation (62, 63).

The related ATP7B (or the Wilson Disease protein) also delivers copper to the trans-Golgi network and is the predominant copper pump in hepatocytes (liver cells). Like ATP7A, ATP7B has six metal-binding domains (MBDs) at the N terminus designated MBD1 to MBD6. Each MBD involves 70 amino acid residue repeated unit that binds one reduced copper ion (Cu^+^) in a Cys-x-x-Cys motif. Among the MBDs, MBD5 and MBD6 regulate the affinity for copper at the metal-binding site in the membrane (64). In structures of ATP7B, MBD1 to MBD4 are not ordered, but MBD5 and MBD6 form a tight unit close to the first transmembrane segment (65, 66). Like ATP7A, ATP7B undergoes regulated trafficking in a way that involves a terminal domain. Upon elevated copper ATP7B traffics to the endo-lysosomal compartment and from here to the apical/canalicular membrane where it drives the efflux of copper into the bile. Apical targeting of ATP7B depends on a nine amino acid-residue targeting determinant in the disordered N-terminal peptide preceding MBD1 (67, 68).

The copper chaperone Atox1 delivers copper to ATP7B but also has a regulatory role. Thus, ATP hydrolysis is activated when Atox1 interacts with the first three MBDs (MBD1 to MBD3) (69). Based on mutagenesis work, it was proposed these MBDs receive copper cooperatively from Atox1, and when MBD3 is loaded with copper an inhibitory interaction is relieved (70). This would suggest that MBD1 to MBD3 serve an autoinhibitory function that is neutralized following sensing of copper.

Another mechanism in P1B ATPases that couples metal sensing with surface expression is observed in the cadmium pump Pca1p in the yeast *S. cerevisiae*. This pump is constitutively synthesized at the endoplasmic reticulum, but immediately becomes ubiquitinated at the N-terminus, delivered to the proteasome and rapidly degraded (71). However, the N-terminal domain also binds cadmium. Following binding of cadmium, the degradation signal that dictates ubiquitination is masked, the protein is stabilized and delivery to the plasma membrane is now possible (72). Most likely, the binding of cadmium to the cysteine-rich N-terminal domain causes a structural rearrangement that hides the ubiquitination site (73). Thus, the N-terminus serves as a cadmium sensor that controls the delivery of the pump to the plasma membrane.

Strikingly, the cadmium-binding region in the N-terminal domain of Pca1p does not bind zinc (73). This is surprising as cadmium is a zinc mimic in biological systems. Thus, it is a common characteristic of zinc-transporting P1B-2 ATPases that they also transport cadmium (74). It may be that the transport machinery of Pca1p itself cannot distinguish between zinc and cadmium, whereas the N-terminal domain fine-tunes specificity. Could it possibly be that in Pca1p—and in other P1B ATPases as well—one function of the metal-binding terminal domain(s) is to serve as selectivity filters that first bind the metal ion to be transported and then direct it to the transport site?

In the model plant *Arabidopsis thaliana*, two P1B-2 zinc pumps, HMA2 and HMA4, deliver zinc from roots to shoots and from there to the developing seed. The C-terminal domain of HMA4 comprises 13 cysteine pairs and a terminal stretch of 11 histidines and serves as a high-capacity chelator (sensor) of zinc that can bind at least 10 zinc ions (75). Apparently, only the di-cysteine motifs but not the histidine stretch contribute to high-affinity zinc binding (76). Full-length HMA4 cannot complement a zinc-sensitive strain of yeast, but sequential deletions of the C-terminal domain result in a progressive increase in Zn^2+^ and Cd^2+^ tolerance and lowered Zn^2+^ and Cd^2+^ content of transformed yeast cells. Deletion of the C-terminal domain *in planta* interferes with the targeting of HMA4 to the plasma membrane, which suggests that plant plasma membrane targeting signals reside within the domain (76).

In conclusion, it seems that N- and/or C-terminal cytoplasmic domains of several eukaryotic P1B ATPases can serve at least four functions: as ligand receptors, as trafficking determinants, as specificity determinants, and as autoinhibitors. It has already been hypothesized that these domains serve as metal sensors that dictate the pump cellular destination (77). The question is, should eukaryotic P1B ATPases as such be classified more broadly as transceptors (transporters that can also serve as receptors; examples are given in ref. (78))? And—since prokaryotic sequences do not have such terminal domains—when did these functions develop?

## P2A ATPases: why two subfamilies?

P2A ATPases are Ca^2+^ pumps and perhaps the best studied of all P-type ATPases (79, 80). P2A ATPases likely evolved to keep cytoplasmic Ca^2+^ low so that it does not precipitate cellular inorganic phosphate (81), which, amongst other functions, is essential for the synthesis of ATP, DNA, and RNA. In humans, a major function is also to transport Ca^2+^ into the sarcoplasmic reticulum to allow for muscle relaxation during exercise (82).

The P2A ATPase SERCA1 was the first P-type ATPase for which a crystal structure was solved (25) and now more than 55 structures are deposited in the protein database. Combined with extensive mutagenesis evidence (83) this implies that the structure/function relationship and mechanism of transport of these pumps now are known in uttermost detail (2, 83, 84, 85, 86).

All prokaryotic P2A ATPases group in a single monophyletic family whereas a gene duplication occurred in the last eukaryotic common ancestor (LECA) giving rise to two subfamilies of eukaryotic P2A ATPases, P2A-I and P2A-II with the canonical SERCA1 belonging to the P2A-I ATPase subfamily (87). The P2A-II ATPases (Fig. 3*A*, #148 - #152) differ from P2A-I ATPases (Fig. 3*A*, # 137 - #147) by having a single residue insert (after Gly721 in helix 7 of the P domain) in a region that interacts with the A-domain to make a pivot for rotation of the A-domain (88) and by having a double proline motif in TM6, a transmembrane segment that contributes to coordination of both of the Ca^2+^ ions transported (87). The subfamilies are not well separated in the phylogenetic tree, which is based on core sequences only (Fig. 3*A*). Most species of green plants (Chloroplastida: land plants and green algae) and the clade Stramenopiles have retained members of both subfamilies, whereas animals and fungi have lost P2A-II ATPases and only have P2A-I ATPases (87). In the model plant *A. thaliana*, endoplasmic reticulum Ca^2+^-ATPase3 (ECA3) is a P2A-I ATPase whereas ECA1, ECA2, and ECA4 are P2A-II ATPases. Species in the clade Alveolata have lost P2A-I ATPases and only have P2A-II ATPases.

Nothing is known about the functional consequences of these strictly conserved changes in the sequence of P2A ATPases. However, although the functional significance of having two subfamilies of P2A ATPases remains mysterious, it is striking that these families have remained side by side since the origin of eukaryotes.

## P2B ATPases: did regulatory domains evolve independently?

P2B Ca^2+^-ATPases resemble P2A ATPases in mechanism but are special by being calmodulin-activated pumps important for cellular signaling (89, 90, 91). A terminal cytoplasmic portion of the pumps serves as a regulatory (R) domain that binds calmodulin and functions as an auto-inhibitor of pump activity.

Autoinhibition is caused by binding of the R domain to the catalytic machinery (92, 93) but details are not known as the R domain is not visible in the so far only structure of a P2B ATPase (the human plasma membrane Ca^2+^-ATPase1; PMCA1) (94). Calmodulin binds to the autoinhibitory domain but only in the presence of Ca^2+^. Thus, the internal docking site and calmodulin compete for binding to the autoinhibitory domain; in the absence of Ca^2+^, the docking site wins, and in the presence of Ca^2+^, calmodulin wins (95). In the plant *A. thaliana* and in humans, the R domain has two calmodulin binding sites with different affinities (96). Mathematical modeling, which is supported by experimental evidence, has revealed that this results in a very steep activation curve when a given threshold of cytoplasmic Ca^2+^ is passed (96).

Strikingly, the R domain of animal P2B ATPases is an N-terminal extension, and that of plants is a C-terminal extension (97). Since the domains originate from opposite sides of the pump molecule, they are for sterical reasons unlikely to interact with the same docking region. However, both have two calmodulin sites in their domains and respond with about the same affinity to a rise in cytoplasmic Ca^2+^. This raises the question as to whether the R domain of plants and animals, respectively, were swapped between ancestral pumps or, alternatively, they evolved independently? As the R domains of plants and animals show very little similarity (98), the latter possibility appears most likely. Interestingly, in P2B ATPases, the calmodulin-binding domain in animals evolved with the emergence of multicellularity, whereas in plants its appearance coincides with terrestrialization (99). This would suggest that the calmodulin-binding domain of P2B ATPases, which allows for accurate Ca^2+^ control and signaling, evolved as a new regulatory layer required for multicellular life in challenging environments.

## P2C ATPases: what do they do in archaea?

The Na^+^/K^+^-ATPase is a marvelous pump that at the expense of a single ATP pumps out three sodium ions and imports two potassium ions (100). In this way, it maintains the Na^+^ gradient and membrane potential that is the basis for the essential biophysics of animal cells.

Why does the Na^+^/K^+^-ATPase have such an elaborate stoichiometry? To generate a membrane potential, why not just pump out a single Na^+^ and leave K^+^? Or, if K^+^ is also needed by the cell, why not adopt a simple 2 Na^+^: 1 K^+^ stoichiometry? The structure of the Na^+^/K^+^-ATPase with bound Na^+^ may provide the answer (101). This work hypothesizes that it is not just for efficiency that three Na^+^ ions are transported per ATP molecule hydrolyzed, it is also for specificity. The three Na^+^ ions do not bind at the same time but sequentially and cooperatively. Thus, for the first Na^+^ to be able to bind, the other sites must be absent, but when the Na^+^ binds it causes an induced fit that generates the second Na^+^ and so on. The ion binding sites are placed so close to each other that only three Na^+^ but not K^+^ can bind at the same time (101). The free energy available in ATP is not enough to export three electrogenic Na^+^ at the same time at least two positive charges need to be counter-transported, in this case, two K^+^.

The Na^+^/K^+^-ATPase has been considered unique for animals (102, 103) and early phylogenetics based on available sequence data suggested that Na^+^/K^+^-ATPase evolved from P-type H^+^ pumps with the emergence of the animal kingdom (102). However, a recent surprising finding in phylogenomics has therefore been that the Na^+^/K^+^-ATPase could have evolved in methanogenic archaea (104). These organisms are truly ancient as they evolved some 3.5 billion years ago (105). The genomes of many methanogenic archaea harbor sequences that encode for Na^+^/K^+^-ATPase that are almost identical to animal Na^+^/K^+^-ATPases, *i.e.* the amino acid residues that contribute to the three Na^+^ and two K^+^ sites are all conserved in the proteins predicted to be expressed from these genes (104). Still, the products of the archaeal Na^+^/K^+^-ATPase genes have so far not been characterized with respect to function and physiological role, so at present, we can only speculate about their function.

What could be the function of Na^+^/K^+^-ATPase in methanogenic archaea? Methanogens are the only organisms known to simultaneously generate a primary electrochemical H^+^ gradient and a primary electrochemical Na^+^ gradient across the membrane at the same time (106). An electrogenic Na^+^ pump has already been described in methanogenic archaea, the *N*^5^-methyl-tetrahydromethanopterin: Coenzyme M methyltransferase or simply Mtr (107, 108, 109, 110, 111). However, this six-subunit pump, which is present in all methanogenic archaea (112), is not driven by ATP but by a methyl transferase reaction. By using a series of hydrogenases to split four H_2_, the methanogenic archaea can use the eight electrons extracted as reducing equivalents to convert one molecule of CO_2_ to a molecule of CH_4_ with two H_2_O as a side product. This process is strictly Na^+^ dependent. During the process, the carbon derived from CO_2_ is incorporated into an organic donor molecule and becomes progressively hydrogenated. The resulting methyl group is subsequently transferred to an organic acceptor molecule. The methyl transferase reaction is coupled to electrogenic Na^+^ transport out of the cell by a mechanism that remains to be described.

What is the function of generating such a Na^+^ gradient? The synthesis of ATP in the methanogenic archaea *Methanosarcina barkeri* is dependent on the proton motive force, *i.e.* the downhill influx of protons (107). H^+^ is also the coupling ion for ATP synthesis in *Methanosarcina mazei* (113). ATP synthesis is carried out by an A_1_A_O_ ATPase, which resembles prokaryotic F_1_F_O_ ATPases, and occurs when H^+^ is funneled into the cell through this complex. It is unclear whether this enzyme in all methanogens can use Na^+^ as a substitute for H^+^ (112, 113, 114, 115). To explain the role of Na^+^ it has been speculated that the Na^+^ gradient that develops across the membrane drives H^+^ efflux *via* an H^+^/Na^+^ antiporter, in this way converting the Na^+^ gradient to an H^+^ gradient that subsequently can be used for ATP synthesis (116). It could also be hypothesized that another yet undescribed enzyme can utilize the Na^+^ gradient to synthesize ATP directly.

If a Na^+^ gradient is required for ATP synthesis, does it make sense then to have a Na^+^/K^+^-ATPase that splits ATP to generate a Na^+^ gradient? Could it be involved in volume control? Maybe the Na^+^/K^+^-ATPase of methanogenic archaea does not split ATP at all, but rather works in the reverse direction to synthesize ATP (117, 118)? In other words, if the thermodynamic driving force permits it, import of three Na^+^ and export of two K^+^ would in this case result in one molecule of ATP being synthesized. If this model is true, could the Na^+^/K^+^-ATPase possibly be the main enzyme that employs the Na^+^ gradient in methanogenic archaea to directly generate ATP?

## P2D ATPases: why this loose Na^+^ specificity?

P2D ATPases are Na^+^ pumps of fungi and mosses and are required for exporting this ion when the Na^+^ levels get high and toxic (119, 120). However, even though these pumps prefer Na^+^, they also transport K^+^. K^+^ is valuable for fungi and mosses as their major electrolyte, and is present in only low concentrations in the terrestrial environment. It therefore makes sense that P2D ATPases are barely expressed in the absence of Na^+^ (121). However, this loose specificity appears strange. Why are they not more specific for Na^+^ in order not to lose K^+^?

The explanation may be linked to the specificity problem discussed above for the Na^+^/K^+^-ATPase. Concentrations of K^+^ in unfertilized soil solutions are typically in the micromolar range (122) and the Na^+^/K^+^-ATPase requires millimolar concentrations for optimal activity (extracellular K^+^ in animals is 4 mM) (100). For this reason, fungi and mosses may not benefit from a Na^+^/K^+^-ATPase for extrusion of Na^+^ as they do not have access to enough K^+^ (or an alternative non-toxic cation), which therefore becomes a limiting factor.

## P3A ATPases: were they electrogenic from the start?

P3A ATPases are plasma membrane H^+^-ATPases, *i.e.*, proton pumps. They have a simple structure with only a single membrane-embedded polypeptide of about 1000 residues and, in cells of plants and fungi, pump out one H^+^ per ATP split to generate membrane potentials that typically range between −100 and −250 mV. Because of the activity of these pumps, secondary active co-transporter systems in the plasma membrane of these organisms can link an energetically downhill movement of H^+^ to the uphill transport of specific solutes into and out of the cell (91, 123, 124, 125). Thus, they are counterparts of the Na^+^/K^+^-ATPase in animal systems but use H^+^ instead of Na^+^ as the energy currency and generate much higher membrane potentials (−60 mV is a typical value for membrane potentials in animal cells).

Prokaryotic cell membranes are also energized by H^+^, but the responsible transporters are not P-type ATPases, but rather multi-subunit protein complexes found in the electron transport chain of the cell membrane and energized by redox processes. H^+^ that amasses in the periplasmic space between the cell membrane and the outer protective layer can return to the cell through the ATP synthase complex and during this process drive the synthesis of ATP (126, 127). Neither components of the electron transport chain nor ATPase synthase are evolutionarily related to P-type ATPases. This raises the question as to how eukaryotic plasma membrane H^+^-ATPases evolved and when they acquired their function to energize the plasma membrane?

The P3A ATPases of plants and fungi also function as pH stats to control cytosolic pH. To prevent acidification of the cytosol, the proteins have a relatively narrow pH optimum, and under normal conditions are autoinhibited at cytosolic pH but become strongly activated if the cytosol acidifies (128). In plants, the cytosolic pH is approximately 7 to 7.5 and lower in yeast (pH 6–6.5) and it is therefore not surprising that the pH optimum of the yeast plasma membrane H^+^-ATPase (about pH 5.5) is lower compared to the plant plasma membrane H^+^-ATPase (about pH 6.5). In plant and fungal P3A ATPases, the H^+^ acceptor/donor in TM6 is a conserved aspartate residue (Asp-684 in AHA2; Asp-730 in *Neurospora crassa* Pma1), the p*K*_a_ of which is likely to be controlled by a single arginine residue in TM5 (Arg-655 in AHA2; Arg-695 in the *N. crassa* Pma1) (129). In the high-affinity E1 conformation of AHA2, the positive charge of the arginine is quite distant from the protonated aspartate, while in the E2 conformation, it is predicted to move closer and establish a salt bridge with the aspartate with the expulsion of H^+^ as a result (36). The arginine residue in TM5 of fungal and plant ATPases is not at the same position in the membrane segment (130), which may explain the different pH optima.

Prokaryotic P3A ATPases are mainly found in methanogenic Archaea but their transport function remains to be determined (103, 131). In contrast to prokaryotic P3A ATPases, fungal and plant P3A ATPases have extended N- and C-termini, which serve as regulatory domains (132, 133). The C-terminal regulatory domains of fungal and plant P3A ATPases have no homology and are of very different lengths, which suggests that they evolved independently (134). Interestingly, the evolution of the C-terminal regulatory domain of plant P3A ATPases coincided with the transition of plants from water to land (134). Mutants of *A. thaliana* that encode C-terminally truncated versions of the P3A ATPase AHA2 grow faster but cannot control water loss and are more susceptible to pathogen infections. This suggests that tight control by the R domain of P3A ATPase activity is essential for the survival of plants in a terrestrial environment (134). Broadening of the function of the pump from simple pH regulation to energization of the plasma membrane may thus have required the establishment of a new regulatory level to control pump activity.

The structures of the plasma membrane H^+^-ATPases from the fungi *S. cerevisiae* and *N. crassa* demonstrate that the proteins are arranged in hexameric rings surrounding a highly ordered array of lipid molecules (135). In the hexamer, the R domain of one pump unit makes contact with the catalytic machinery of the neighboring unit and, in this way, each pump unit is controlling its neighbor. In the activated state, the plasma membrane H^+^-ATPase remains in a hexamer (135). The plant plasma membrane H^+^-ATPases isolated in the monomeric state is an active protein (136). However, there is evidence accumulating that plant plasma membrane H^+^-ATPases may function in dimers with the R domains reciprocally controlling the neighboring protein unit (137). In plants, protein kinase-mediated phosphorylation of the penultimate residue in the C-terminal end of the plasma membrane H^+^-ATPase creates a binding site for 14-3-3 protein, which upon binding neutralizes pump autoinhibition (138, 139, 140). The 14-3-3 protein/plasma membrane H^+^-ATPase complex observed by cryo-electron microscopy has a wheel-shaped structure with six-fold symmetry, which suggests that in the hyperactivated state, the dimers of plasma membrane H^+^-ATPase become organized into a ring comprised of three pump dimers possibly united by three 14-3-3 protein dimers (141).

An unresolved question related to the transport mechanism of plasma membrane H^+^-ATPases is the nature of the bound ligand. It is well established that the conserved aspartate in TM6 serves as an H^+^ acceptor (36, 129), but as in other P-type ATPases, TM4 is unwound to create the canonical ion binding site in the center of the membrane domain. As the hydronium ion (H_3_O^+^) has an ionic radius corresponding to that of Na^+^, it has been suggested that a water molecule is coordinated at the cavity formed by TM4 and shares an H^+^ with the carboxyl group of the aspartate (142). The structures of P3A ATPases solved so far are of a resolution that is not sufficient to identify water molecules. Thus, the exact mechanism of H^+^ transport and whether water is also transported will have to await high-resolution structures of plasma membrane H^+^-ATPases. An alternative model, that does not involve a water molecule, has proposed a direct capture of the H^+^ in a hydrogen bond between the aspartate and an opposing conserved asparagine (Asn-154 in *N. crassa*; Asn-106 in AHA2) (36, 130).

## P3B ATPases: are they really Mg^2+^ pumps?

P3B ATPases group phylogenetically with plasma membrane H^+^-ATPases (P3A pumps) but since their discovery in *Salmonella enterica* serovar Typhimurium they have been linked genetically to Mg^2+^ transport.

*S. enterica* is a facultative intracellular pathogen that may experience Mg^2+^ deficiency inside cells such as macrophages, where they reside in an acidic nutrient-poor endomembrane compartment, the phagosome (143). The infected mammalian cell recruits Natural resistance-associated macrophage protein 1 (Nramp1, SLC11A1) to the phagosome membrane where it functions as a secondary active proton-coupled divalent metal ion transporter to induce Mg^2+^ starvation of the engulfed *S. enterica* (144). Analysis of mutants deficient in uptake of Mg^2+^ revealed that at least three genetic loci in *S. enterica* are linked Mg^2+^ transport: *corA*, *mgtA*, and *mgtB* (145). Strains with mutations at all three loci are not able to take up Mg^2+^ but can be complemented with a wild-type allele representing any one of the three loci (145).

CorA encodes a Mg^2+^ selective channel protein (146, 147), and appears as the main Mg^2+^ transporter of prokaryotes. In contrast herewith, *mgtA* (148) and *mgtB* (149) both encode P3B ATPases (26), which are not as widely spread within prokaryotes. Transcription of *mgtA* and *mgtB* is repressed by Mg^2+^ but, when the cell senses that Mg^2+^ is limiting, expression of these genes is induced more than a thousand-fold (150, 151, 152). A similar induction of gene expression is seen when cells experience osmotic shock, which causes cell membrane depolarization (153). The Mg^2+^ status is sensed at both the mRNA level, where Mg^2+^ binding to the 5′ untranslated region of *mgtA* represses transcription of the gene (151), and at the protein level, where high Mg^2+^ inhibits protein function (153). Purified MgtA protein is stimulated by micromolar concentrations of free Mg^2+^ but its affinity appeared to be independent of H^+^ in contrast to what would have been expected if H^+^ is counter-transported in exchange of Mg^2+^ (154). As influx of Mg^2+^ through CorA is normally downhill an electrical gradient, which would not be expected to require a primary active transporter, it has been suggested that ATP-driven uptake of Mg^2+^ by MgtA and MgtB is required under conditions when the membrane potential is reduced or even reverted (positive inside), a condition that is found in prokaryotes living in acidic environments (155, 156).

However, doubts about the function of MgtA and MgtB as just a supplement to the activity of CorA were expressed by Maguire and coworkers already in 1998 (150). First, when *S. enterica* was cultivated at pH 5.2, the CorA-mediated uptake of Mg^2+^ was not inhibited (in fact, uptake was better compared to the capacity at pH 7.4). Second, the induction of *mgtA* and *mgtB* by Mg^2+^ deficiency was stronger in the presence of CorA than in its absence. Third, induction of *mgtA* by low Mg^2+^ was almost lost at pH 5.2 compared to induction at pH 7.4 whereas induction of *mgtB* was reduced to a lesser extent. Fourth, the ability of *S. enterica* to invade and survive within host cells was not affected by the lack of MgtA and MgtB (150). Taken together, this was taken as evidence that inside a phagosome the CorA system would be sufficient for Mg^2+^ acquisition. The authors concluded “the overall data indicate that Mg^2+^ induction of *mgtA* and/or *mgtB* expression is not (solely) for the purpose of increasing Mg^2+^ uptake capacity” and it was suggested that MgtA and MgtB have additional functions besides transporting Mg^2+^.

P3B ATPases are not only restricted to prokaryotes but have a surprising presence in several land plants (157). As no other eukaryotic kingdoms harbor P3B ATPases (and they are also absent from green algae, the nearest relatives of land plants), their presence in land plants may be the result of horizontal gene transfer (157). In plants, P3B ATPases appear to play a role in the acidification of vacuoles. The vacuoles of plants are acidified by V-type ATPases and H^+^ pyrophosphatases but in addition, the plant vacuolar membrane harbors a P3A-type plasma membrane H^+^-ATPase (AHA10 in the model plant *A. thaliana*), which contributes to acidification of the vacuolar lumen (158, 159). In the petals of petunia (*Petunia hybrida*) flowers, the activity of PH5, an AHA10 homolog, determines the pH-dependent color of flower pigments in the vacuole (160). PH5 physically interacts with a P3B ATPase, PH1 (161). Apparently, PH1 has no H^+^ transport activity on its own but enhances the transport the H^+^ transport capacity of PH5. Thus, it was suggested, that the hyperacidification of vacuoles in petals requires a heteromeric P-ATPase pump comprising both a P3A and a P3B ATPase (161). The extreme acidity of *Citrus* fruits resulting from a vacuolar pH of around pH 2 is likewise determined by the combined action of PH1 and PH5 homologs (162) and the acidity of apple fruits also correlates with the expression of a P3B ATPase (*Ma10*) (163).

Could plant P3B ATPases possibly be Mg^2+^ transporters? If they function in a similar way to what has been proposed for MgtA and MgtB, they would be expected to import Mg^2+^ from the extracytosolic side (*i.e.* the vacuolar lumen) to the cytosol. This seems to make no sense, as the vacuole is surrounded by a membrane with an outside negative membrane potential, and therefore a channel protein should be sufficient to release Mg^2+^ from the vacuole.

Could it be that MgtA and MgtB are in fact H^+^-ATPases, or regulate H^+^-ATPases? In such a proposed role, they could serve to assist *S. enterica* in extruding H^+^ when it experiences acidic environments and contribute to re-establishing the membrane potential following membrane depolarization. Sensing of low Mg^2+^ could be the signal that reports that the pathogen has entered a eukaryotic cell and needs an additional component to energize the cell membrane. This would explain the link between Mg^2+^ limitation and induced expression of *mgtA* and *mgtB*. P3B ATPases have a conserved Asp residue in TM6, which corresponds to the H^+^ acceptor/donor in P3A ATPases (Asp-684 in AHA2) in P3A ATPases. Arguing against P3B ATPases being directly involved in H^+^ translocation is that they lack the Arg residue in TM5 (Arg-655 in AHA2) that controls the H^+^ affinity of the residue corresponding to Arg-684 in AHA2 (154).

## P4A ATPases: how did lipid flippases evolve?

P4 ATPases are unique for eukaryotes and are involved in flipping phospholipids and ceramide-derived lipids from the extracytosolic side of biological membranes to their cytosolic side. They are mainly expressed in the secretory pathway system (except for the endoplasmic reticulum but including the plasma membrane), where they generate membrane lipid asymmetry and contribute to vesicle formation (164, 165). Phylogenetically, they are divided into three subfamilies: P4A to P4C (166).

**Figure 6:**
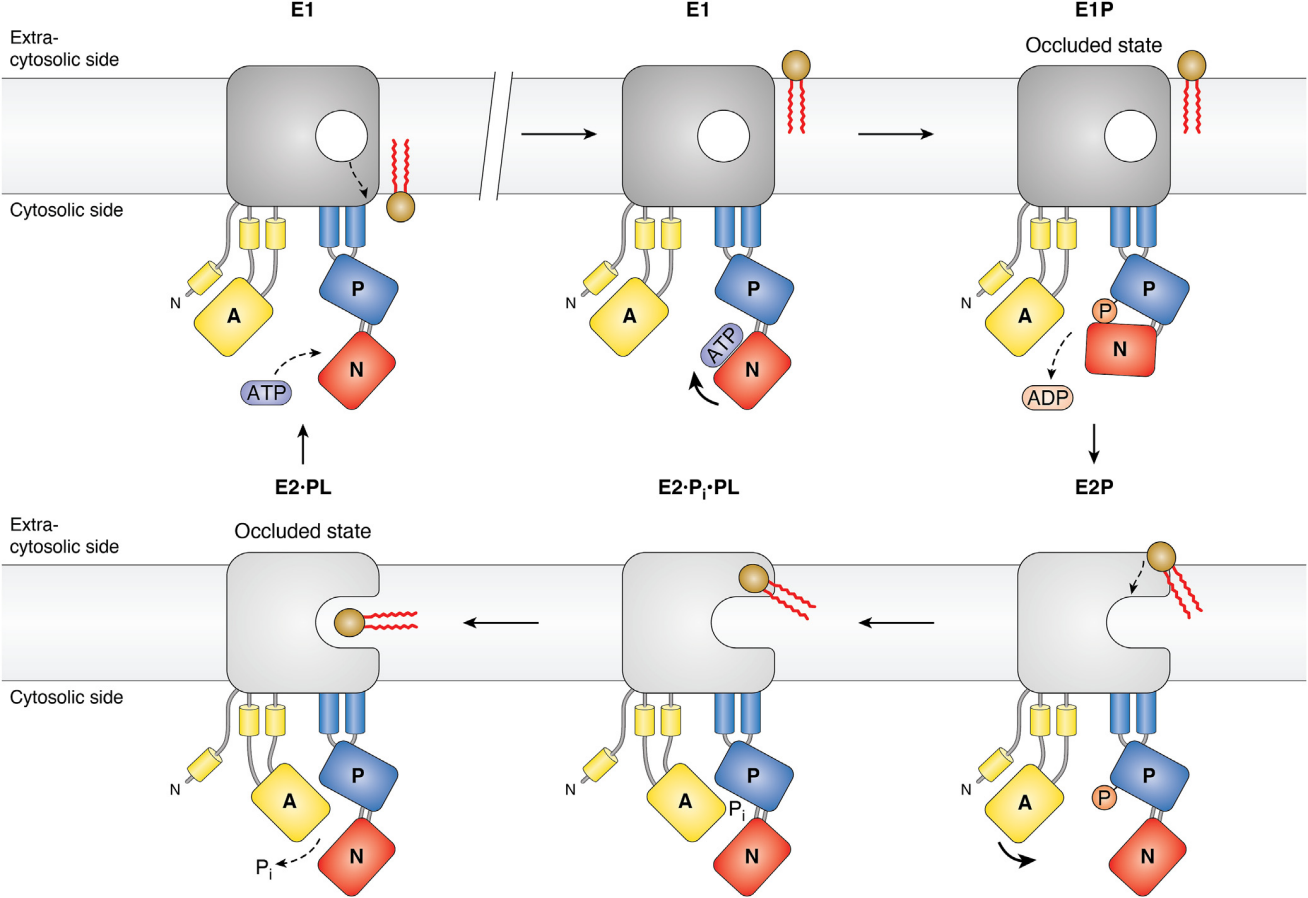
**Variations on a theme: the central cavity model of P4A ATPases.** P4A ATPases flip phospholipids from the extra-cytosolic side of membranes to the cytosolic side. Before transport, the E1 conformation of the pump is spontaneously phosphorylated from ATP. Pump phosphorylation results in the transition to the E2P conformation, to which the phospholipid head group binds. Binding of the phospholipid induces pump dephosphorylation and the phospholipid then slides through a cleft in the membrane domain toward the canonical binding site. In the dephosphorylated E2 conformation, the phospholipid is occluded in the membrane domain with its headgroup positioned in the CBS and its acyl chains protruding out of the protein. During the E2P to E1 transition, the phospholipid is released to the intracellular leaflet of the bilayer.

For a long time, it remained a mystery how a giant substrate like a phospholipid could be transported through the lipid bilayer by a P-type ATPase, which is intriguing considering that most members of this superfamily transport much smaller cations. Pomorski and Menon (167) proposed a model for phospholipid transport across the membrane, which was likened to that of swiping a card through a card reader. According to this “credit card model,” the polar headgroup of the phospholipid slides through a groove in the P4 ATPase molecule from one side of the membrane to the other while its acyl chains remain in the membrane. The model was subsequently revised (168, 169, 170, 171) based on mutagenesis data, which suggested that the phospholipid head group interacts with a central cavity in the protein like cations do in cation-transporting P-type ATPases (172). According to the “central cavity model” (Fig. 6), the headgroup is not sliding in a single motion through the membrane (168, 173). Rather, halfway through the membrane it pauses and is occluded in a cavity (the canonical binding site; Fig. 1*A*) that involves coordination by carbonyl oxygens in TM4. An unfolded TM4 giving room for a cavity in the membrane domain was indeed observed in the first structure of a P4 ATPase (169). Following dephosphorylation, a conformational change allows the phospholipid headgroup to be released and an exit groove is now available to allow for further passage through the membrane (173). Structural studies of P4A ATPases in different conformations support the presence of the occluded state (170, 174).

It has been noted that the β subunit of P4A ATPase resembles the combined β and γ subunits of Na^+^/K^+^-ATPase but the subunits of the two pumps are not evolutionarily related (175). P4A ATPases are associated with a β subunit (also called the Cdc50 protein), which has 2 TM helices and a large luminal loop that is decorated with sugar residues. In the case of the Na^+^/K^+^-ATPase, the β subunit has a single TM segment followed by a large luminal glycosylated loop and, in addition, may be associated with a small TM spanning γ subunit (or FXYD protein) providing an additional TM segment (137). The β subunit of the P4A ATPase assists in protein folding in the ER and is required for ER exit. In this way, it functionally resembles the β subunit of mammalian Na^+^/K^+^-ATPase, which is also required for proper folding and exit from the ER (176). However, despite this analogy, there are also large differences in the function of these subunits. The P4A ATPase β subunit makes multiple contacts with the P4A ATPase on both the luminal and cytoplasmic side of the membrane and within the membrane. This tight grip restricts the conformational flexibility of the membrane domain (170, 177, 178), which possibly is key to the transport function of P4A ATPases (173). The β and γ subunits of the Na^+^/K^+^-ATPase do not put similar constraints on the membrane domain.

The function of P4B ATPases has long remained a mystery. P4B ATPases are unique in that they do not associate with a β subunit. The yeast *Saccharomyces cerevisiae* has a single P4B ATPase, Neo1p, which is essential for growth and is part of a membrane remodeling complex in the Golgi apparatus (179, 180). The Neo1p protein has been purified and although transport was not shown directly, it could be demonstrated to have an ATP hydrolytic activity that was stimulated by phosphatidylethanolamine, phosphatidylserine, and lyso-phosphatidylserine with the highest affinity for the latter (181). Further investigation is required to ascertain whether these lipids directly function as substrates for Neo1p or if they act as co-factors influencing its activity. Scaffold functions independent of lipid flippase activity have been reported for P4A ATPases (182). Intriguingly, therefore, there is a possibility that P4B ATPases, instead of serving as lipid flippases, may have a lipid transport-independent function as scaffold switches to recruit structural components or effectors.

The Neo1p structure was solved in three different conformations and turned out to be very similar to that of P4A ATPases except for three luminal loops, which in P4A ATPases make tight contact with the β subunit (183), but in Neo1p are much shorter (181). This has led to the proposal that P4A ATPases may have evolved from P4B ATPases by extending their luminal loops to allow for tight interaction with a β subunit (181).

P4B ATPases are absent from plants that instead express P4C ATPases (ALA2 in the model plant *A. thaliana*), which are phylogenetically related to P4B ATPases but associate with a β subunit (166). P4B and P4C ATPases have representatives in both unikonts (including Metazoa and Fungi) and bikonts (including Plantae and Alveolata amongst others), which suggests that both have an ancient origin and evolved before the split into the eukaryotic kingdoms known today (166). Thus, P4C ATPases may represent an evolutionary transition from P4B ATPases to P4A ATPases.

## P5A ATPases: are they transmembrane helix flippases?

P5 ATPases, like P4 ATPases, are unique to eukaryotes where they are omnipresent and have no resemblance with P-type ATPases of prokaryotes. They were first identified from gene sequences when eukaryotic genomes started to become available and described as P-type ATPases with no apparent specificity (26), and they remained orphan pumps until very recently. They are divided phylogenetically into two subfamilies, P5A and P5B (184, 185).

The exact function of P5A ATPases did for a long time remain a mystery, but clarification of their role is now emerging. P5A ATPases are characterized by a negatively charged motif in the putative transport binding site in M4 (PP[D/E]LPxE) and compared to other P-type ATPases have two extra transmembrane helices in the N-terminal region (Ma and Mb) (185). P5A ATPases are encoded for in the genome of all eukaryotic cells. Typically, only a single gene is present in the genome of each species and the expressed protein localizes to the endoplasmic reticulum (ER). Loss-of-function mutations in the gene result in severe ER stress and complex pleiotropic phenotypes are observed (186). This suggests that a P5A ATPases is responsible for a very basal function in the ER and in its absence, the functions of multiple proteins are compromised.

Some of the phenotypes of the *S. cerevisiae* P5A ATPase Spf1p illustrate this point. The gene was first identified in a screen for mutants resistant to *Pichia farinosa* killer toxin, hence its name Sensitivity to *Pichia farinosa* (187). In the mutant, the toxin is not internalized and defects in the glycosylation of cell wall components were observed (187). In a second genetic screen, *spf1* was identified as being unable to correct the topology of a misoriented reporter membrane protein in the ER (188). Subsequently, in a screen for yeast mutants that are highly amenable for transformation, which involved about 5000 yeast knockout strains, *spf1* was one of the three most transformation-competent mutants identified (189). Then, in 2012, a yeast genetic screen was carried out to identify loss-of-function mutants that accumulate mitochondrial tail-anchored proteins in the ER. The only mutant identified with this phenotype was deficient in *SPF1* (190).

The mechanism of P5 ATPases was first attempted to be understood by finding a common denominator that could be responsible for the large number of seemingly unrelated phenotypes of P5A ATPase mutants. For example, in the absence of Spf1p, ergosterol metabolism is disturbed and ergosterol levels increase in the ER (190, 191). Since sterols influence membrane properties, an altered sterol composition of the ER would influence the activity of membrane proteins and could possibly explain many effects resulting from the loss of a P5A pump (191).

The breakthrough work of McKenna *et al*. (192) elucidated the structure of Spf1p and became a major step in our understanding of P5A function. It demonstrated that tail-anchored proteins destined for the mitochondrial outer membrane interact physically with Spf1p. Furthermore, the structure of Spf1p revealed a deep binding pocket with negative charges donated by TM4 in which a density resembling an alpha helix was present. This was interpreted as being the transmembrane segment of a tail-anchored protein.

In this context, the stellar paper by Tipper and Harley (188) provides important insight. In prokaryotes, positively charged loops of membrane proteins of newly synthesized membrane proteins end up on the cytoplasmic side of the cell membrane due to the inside negative membrane potential (they follow the so-called “positive-inside rule”; ref. (193)). In eukaryotes, positively charged loops of membrane proteins likewise end up on the cytoplasmic side of the ER membrane despite the absence of membrane potential. Thus, how eukaryotes manage to control topology of membrane proteins according to the “positive-inside-rule” has remained a mystery. Tipper and Harley (188) carried out a screen for yeast mutants that failed to correct the topology of mis-inserted membrane proteins in the ER. This screen was designed to identify mutants in which the positively charged end of a single-spanning membrane protein ended up on the luminal side of the ER. In their screen, they only identified two such mutants, *spf1* and *ste24*.

When the results of Tipper and Harley (188) are considered in light of the structure of Spf1p (192), it becomes evident that Spf1p may function to recognize positive charges of membrane helices or loops exposed on the luminal side of the ER and subsequently send them back to the cytoplasmic side. To recognize a positive charge at the wrong side of the membrane, P5A ATPases would be predicted to have a negatively charged binding pocket facing the luminal side of the membrane, which is in accordance with the structure of Spf1p (192). According to this model, P5A ATPases would not only control the insertion of tail-anchored proteins destined for mitochondria but be involved in the quality control of membrane protein topology as such. Indeed, recent studies have demonstrated that P5A ATPases can control the topology of non-mitochondrial membrane proteins (194, 195, 196, 197).

Following this hypothesis, P5A ATPases are not just ATP-dependent membrane helix dislocases but should be termed transmembrane helix flippases. In this scenario, ALL membrane proteins are potential interactors of the P5A ATPase. The interactome would account for 30% of all proteins in the cell, which would also explain why such a diverse range of phenotypes has been observed in the absence of P5A ATPase function. Such a broad function of P5A ATPases readily explains why its absence result in so diverse phenotypes seemingly not restricted to mitochondrial function.

What is lacking to confirm this model are structural studies of P5A ATPases in complex with identifiable clients. What is also needed is biochemical studies that link ATP hydrolysis to correction of transmembrane helix topology. All P-type ATPases characterized so far are strictly coupled, *i.e.* the hydrolysis of ATP is coupled to vectorial transport. Similarly, does ATP hydrolysis of P5A ATPases require the binding of a client with the wrong topology? If this is the case, why do purified Spf1p preparations have ATP hydrolytic activity (198)? Can this possibly be explained by the presence of contaminating substrates in the preparation or, alternatively, uncoupled activity?

## P5B ATPases: do they transport more than just polyamines?

P5B ATPases are characterized by a conserved Pro-Pro-Ala-Leu-Pro (PPALP) motif in TM4. These pumps have been lost in the plant lineage whereas they have undergone gene expansion in the animal lineage (184, 199). In mammalian cells, four isoforms (ATP13A2-5) are found that are mainly expressed in endo/lysosomes. Further, their dysfunction is linked to cancer (ATP13A2-4), neurodegeneration such as Kufor-Rakeb Syndrome, a form of early-onset Parkinson’s disease with dementia (ATP13A2), cardiovascular diseases such as pulmonary arterial hypertension (ATP13A3), and neurodevelopmental disorders like schizophrenia and autism (ATP13A4) (200).

P5B ATPases were identified as polyamine transporters in 2020 (201) and strict coupling between ATP hydrolysis and polyamine transport has been demonstrated (201, 202). The first structures of ATP13A2 (203, 204, 205) and its homolog in yeast, Ypk9p (206), appeared almost simultaneously in 2021 and more recently structures of ATP13A2 in six different conformations were solved and depict a nearly complete transport pathway (207). In the E2P state, a polyamine is observed in an entry site on the luminal side of the protein, and in the E2-P_i_ state (in which the phosphate bond to the aspartate residue is broken but inorganic phosphate is still bound to the pump) a density likely to represent a polyamine is found within a central cavity of the protein (207). Like in other P-type ATPases, the cavity involves TM4, which is unwound by the conserved PPALP motif. In a putative dephosphorylated E2 state, a density likely to represent the substrate is observed in a deep groove on the cytosolic side of the protein (207).

Surprisingly, therefore, it has been suggested from membrane vesicle studies that the human P5B ATPase ATP13A2 functions as a lysosomal H^+^/K^+^-ATPase (208). Several P-type ATPases use H^+^ as a counterion and it is an intriguing possibility that by serving as an H^+^ pump ATP13A2 would serve to assist in the acidification of the lysosome. Testing this hypothesis will require further work as H^+^ transport was not demonstrated directly. It would appear counterintuitive if K^+^ is transported together with a polyamine which is positively charged already at physiological pH. Unfortunately, as the study was not confirmed in the presence of polyamines, it remains uncertain whether K^+^ is co-transported together with polyamines, or whether K^+^ and polyamines are competing for the same binding site. Studies on the purified protein should help answer these questions.

## P6 ATPases: A new family

Since the classification of P-type ATPases in 1998 (26), sequencing of hundreds of eukaryotic genomes has identified thousands of P-type ATPase sequences, but all belong to one of the P1 to P5 ATPase families. In prokaryotes, however, there is still undescribed diversity (209).

In the phylogenetic tree (Fig. 3*A*), a single sequence (#19; *Mycobacterium tuberculosis* CtpE) stood out and could not be assigned to any family. The CtpE ATPase is not unique for *Mycobacterium* as homologs have been identified in Actinobacteria, Firmicutes, Cyanobacteria, “Candidatus Saccharibacteria”, Chlorobi, Chloroflexi, Planctomycetes, Proteobacteria, and Tenericutes (209, 210). In a phylogenetic tree together with other P-type ATPases these sequences form a distinct clade with strong statistical support at the node (210). It thus seems fair to characterize CtpE and its homologs as a distinct P-type ATPase family. It is here proposed that this new family of P-type ATPases be denoted P6 ATPases.

CtpE was functionally characterized in 2017 and shown to be a Ca^2+^ uptake system, that is only expressed under Ca^2+^ deficient conditions (210). Ca^2+^ concentrations outside cells are normally much higher than in the cytoplasm so it was surprising that a transporter should require energy in the form of ATP to import Ca^2+^. However, *M. tuberculosis* proliferates inside macrophages where free Ca^2+^ concentrations are extremely low (211, 212). Therefore, primary active transport of Ca^2+^ influx is required to survive in this environment.

The question remains, how comes that the transport direction is reverted in P6 ATPases compared to Ca^2+^ efflux ATPases? CtpE and other P6 ATPases contain the PEGL motif in TM4 that is characteristic for Ca^2+^ efflux pumps but lacks the aspartate residue that in Ca^2+^ efflux pumps is omnipresent in TM6 and is involved in coordinating Ca^2+^. Instead, in CtpE, it is replaced by isoleucine, a residue having a bulky hydrophobic side chain. Could this be the key to understand the reversal of Ca^2+^ transport?

## Are there more families and what do they do?

Two other *M. tuberculosis* P-type ATPases have an isolated position in the tree (Fig. 3*A*, #158 and #159; CtpI and CtpH). These have so far not been characterized and it is therefore not known whether they are functional pumps with a distinct substrate specificity. If they are, they might constitute a unique family. Thus, whereas new families in eukaryotes are unlikely to appear from further genome sequencing, prokaryotic diversity is so large that more families are likely to be characterized in this domain.

## Conclusion

P-type ATPases have been described mechanistically in detail, hundreds of structures from all major families and subfamilies are deposited in protein databanks, and the physiological functions of each family are well characterized in multiple organisms from most kingdoms of life. Nonetheless, many enigmas remain, and other researchers in the field would probably have highlighted a different set of unsolved problems than the ones listed in this review. This is only good news: the field is brimming with untapped research opportunities and far from having run its course.

## Conflict of interest

The author declares to have no conflicts of interest with the contents of this article.
